# Increased frequency of psoriasis in the families of subjects with childhood Familial Mediterranean Fever

**DOI:** 10.1186/1546-0096-12-S1-P258

**Published:** 2014-09-17

**Authors:** Kenan Barut, Muruvet Guler, Tugba Erener-Ercan, Metin Sezen, Ozgur Kasapcopur

**Affiliations:** 1 Pediatric Rheumatology, Istanbul University, Cerrahpasa Medical Faculty, Istanbul, Turkey

## Introduction

Familial Mediterranean fever (FMF) is an autosomal recessive disease characterized by periodic fever and polyserositis attacks. FMF can be associated with vasculitis, spondyloarthropaties, Behçet’s disease and inflammatory bowel disease. Psoriasis is another disease that can be associated with FMF. Psoriasis is a common disease affecting approximately 2% of the population. Although there are many clinical subtypes, the most frequent subtype is psoriasis vulgaris. Psoriasis vulgaris comprises 85-90% of all the psoriasis subtypes. The association of FMF with psoriasis has never been investigated so far.

## Objectives

The aim of our study was to investigate the frequency of psoriasis among the family members (mother, father, sibling) and close relatives (uncle, aunt) of children with FMF.

## Methods

The study group consisted of 202 FMF cases diagnosed according to the diagnostic criteria of Yalçınkaya et al, 238 JIA cases diagnosed according to ILAR (*International League of Associations for Rheumatology*) *who were followed up in Istanbul University, Cerrahpasa Medical Faculty*, *Department of Pediatric Rheumatology* and 200 healthy controls. Patients with juvenile psoriatic arthritis in the JIA group were excluded from the study. The presence of psoriasis diagnosed by a dermatologist was questioned among family members and close relatives of all the enrolled cases.

## Results

Psoriasis was detected in 41 (20.3%) of 202 FMF patients (M:25, F:16), 10 (4.2%) of 238 JIA patients (M:3, F:7), 12 (6%) of the family members and close relatives of 200 healthy children. Of the 41 FMF patients with psoriasis, dermatologist diagnosed psoriasis was detected in the mother; in the father; in the sibling; in the grandmother and grandfather; in the aunt and uncle; and in the cousin, in 5, 5,1,11,12,12 of them, respectively. The increased incidence of psoriasis among the parents and close relatives of cases with FMF than that of cases with JIA and healthy controls was statistically significant (p<0,0001).

## Conclusion

The association of FMF with psoriasis has been reported in the medical literature only as case presentations. To our knowledge, this study is the first study investigating this association among FMF patients and their family members. We found an increased incidence of psoriasis in the family members of FMF patients. However, this association should be confirmed with further studies with larger sample sizes and the association of MEFV gene with psoriasis should also be investigated.

## Disclosure of interest

None declared.

